# Peri-articular tranexamic acid injection in total knee arthroplasty: a randomized controlled trial

**DOI:** 10.1186/s12891-016-1176-7

**Published:** 2016-07-26

**Authors:** P Pinsornsak, S Rojanavijitkul, S Chumchuen

**Affiliations:** Department of Orthopaedic Surgery, Thammasat University, 99 Moo 18, Khlong Nueng, Khlong Luang, Pathumthani Thailand 12120

**Keywords:** Tranexamic acid, Peri-articular injection, Total knee arthroplasty, Blood loss

## Abstract

**Background:**

Intravenous tranexamic acid (IV TXA) is one of the most effective agents in use for reducing blood loss following total knee arthroplasty (TKA) but its safety regarding venous thromboembolic events (VTEs) remains in question. The direct, local application of TXA may reduce systemic toxicity whilst maintaining good or better bleeding control compared to IV TXA. The topical application of TXA via Hemovac drains has been reported previously with good results. However, there are no data on peri-articular TXA injections during TKA.

**Methods:**

We conducted an open randomized, pilot study of peri-articular vs. IV TXA in 60 patients undergoing TKA. 30 patients received either: (i) 750 mg peri-articular TXA into the medial, lateral capsules and the quadriceps tendon prior to capsular closure and tourniquet deflation (group1), or (ii) 750 mg of IV TXA just before tourniquet deflation. Blood loss in the hemovac drain and hemoglobin (Hb) concentrations were measured at 24 and 48 h (h), and the number of blood transfusions and leg circumference measurements were recorded.

**Results:**

At 48 h, the total blood loss in the hemovac drain was 445 mL in group 1 vs. 520 mL in group 2 (*p* = 0.081) and the corresponding declines in Hb were 1.85 g/dL vs. 1.87 g/dL (*p* = 0.84). 16 patients received blood transfusions: 9 vs. 7 in groups 1 and 2, respectively (*p* = 0.928). There were no differences in thigh and lower leg circumferences, pain scores, knee flexion at discharge date and lengths of hospital stay. There were no clinically detected venous thromboembolic events.

**Conclusion:**

This pilot study has shown promising results for peri-articular TXA during TKA. Additional, larger studies are needed to confirm our results and be powered to show differences in efficacy and safety of peri-articular vs. IV TXA.

**Trial registration:**

ClinicalTrials.gov Identifier NCT02829346. Retrospectively registered: 07/11/2016.

## Background

Postoperative bleeding in total knee arthroplasty (TKA), which can result in hypovolemic shock and the need for allogenic blood transfusions, is a major concern for orthopedic surgeons. Several strategies are used to reduce postoperative blood loss, including preoperative autologous blood transfusions, hemoglobin raising agents, intraoperative tourniquets, cell savers, intravenous (IV), and topical tranexamic acid (TXA) [1–7]. TXA is an antifibrinolytic agent that inhibits the conversion of plasminogen to plasmin and also acts as a plasmin inhibitor. This results in inhibition of the breaking down of fibrin blood clots (fibrinolysis) [8] and fibrin clot stabilization rather than the promotion of clot aggregation [9, 10]. Plasmin activation due to tissue trauma increases after release of the surgical tourniquet and increased fibrinolysis [8, 11, 12].

IV TXA is commonly used intravenously for reducing blood loss and blood transfusions following TKA. Blood loss reduction varies from 10 % to 70 % compared to control groups not receiving TXA [13–15]. However, there are reports of thrombus formation associated with IV TXA [16, 17]. Topical TXA applications have been reported as another method to reduce blood loss and are associated with fewer systemic side effects compared to IV TXA [6, 7, 18–21]. Topical TXA is usually applied by soaking TXA in the operating field and washed after period of time or left to drain out following wound closure and drain insertion. Topical TXA is only has superficial contact with the bleeding surface and for a limited period of time. We do not know the effect of topical TXA on polyethylene and this needs the further study.

Another approach could be peri-articular TXA injections which would act directly on the injured tissue and for a longer duration compared to topical and IV TXA. This may result in better reduced blood loss comparable or better than topical or IV TXA but without the potential systemic toxicity of IV TXA [22, 23].

Given the lack of data on peri-articular TXA, we evaluated the benefits and toxicity of peri-articular TXA compared to standard IV TXA and report the results, herein.

## Methods

This prospective, randomized trial was conducted from October 2012 to October 2013, and approved by the Ethics Committee, Institutional Review Board of Thammasat University (Registry #MTU-EC-OT-0-096/54). All patients had been provided with written, informed consent prior to their participation in the study.

The study inclusion criteria were adult patients with osteoarthritis in need of a TKA.

The exclusion criteria were the patients with inflammatory arthritis, post-traumatic arthritis, a history of or current venous thromboembolic disease, any underlying disease of haemostasis, cirrhosis, chronic renal failure, patients on anticoagulants or strong antiplatelet drugs (e.g. warfarin, clopidogrel), know allergy to TXA, defective color vision, and a preoperative hemoglobin <10 g/dL or a platelet count < 140,000 /uL^3^.

### Study conduct

**Fig. 1:**
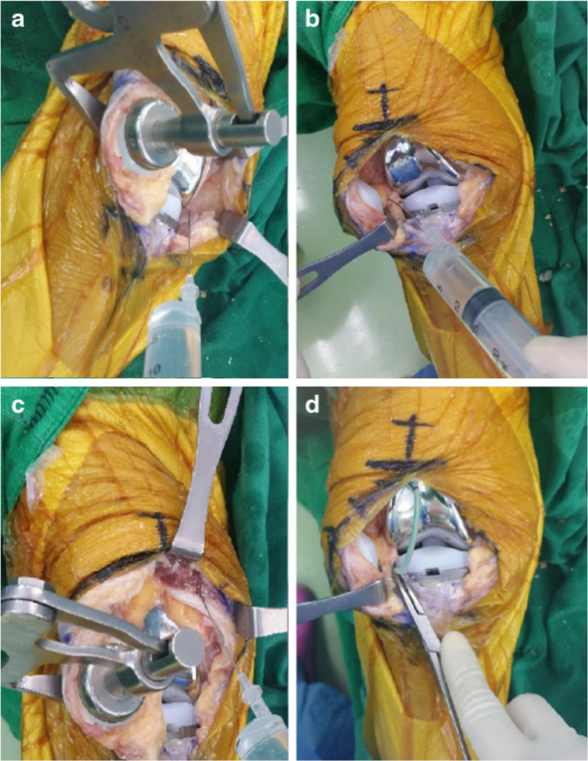
Peri-articular tranexamic acid injection (right knee) at **a** the medial gutter and medial capsule, **b** the lateral gutter and lateral capsule and **c** around the quadriceps muscle. **d** One drain (Privac®; Primed Halberstadt Medizintechnik GmbH, Halberstadt, Germany) was positioned at the lateral gutter

All patients underwent routine preoperative preparations. Patients on aspirin were asked to stop them at least 7 days before the operation. The surgeries were performed by a single surgeon (P.P.). The same surgical technique was used throughout the study. After the tourniquet was inflated, a limited medial parapatellar skin incision (~10 cm length) was made beginning 2 cm proximal to the superior pole of the patella and passing along the medial border of the patella to the medial border of the tibial tubercle. A medial parapatellar approach was used in all cases. Routine femoral (intramedullary guide) and tibial (extramedullary guide) preparations were carried out and the TKAs were performed using the standard measured-resection technique. A cemented, posterior stabilized, fixed bearing TKA prosthesis was used in all cases (Nexgen®; Zimmer, Warsaw, IN, USA). No patellae were resurfaced but were all denervated by electro cautery. Ten minutes prior to deflating the tourniquet, sealed envelopes were opened containing the randomized the allocated treatment. In group 1, patients received 750 mg of peri-articular TXA (Transamin®; OLIC Thailand Ltd, Bangkok, Thailand; 250 mg/5 mL, 15 cc total volume, Fig. 1) injection into the soft tissue around medial capsule (5 ml), lateral capsule (5 ml) and around the quadriceps muscle (5 ml). In group 2, the patients received 750 mg of IV TXA (250 mg/5 ml, 15 cc total volume, keeping within the therapeutic range of 10–15 mg/kg/dose). One drain (Privac®; Primed Halberstadt Medizintechnik GmbH, Halberstadt, Germany) was positioned in the lateral gutter (Fig. 1) and the wound closed. The tourniquet was deflated following skin closure. The drain was clamped and reopened three hours postoperatively. The drain output was recorded at 24 and 48 h (post skin closure) and removed at 48 h in all patients. Hemoglobin and hematocrit levels were recorded at 24 and 48 h postoperatively. The criteria for blood transfusion were a hemoglobin concentration < 10 g/dL or if patients had any signs of anemia (e.g. chest pain that suggested from cardiac origin, congestive heart failure, and unexplained tachycardia or hypotension unresponsive to fluid replacement) at 24 or 48 h. One unit of packed red cells was transfused per 1 g/dL of hemoglobin drop. No anticoagulant agents were used but postoperative foot pump exercises and early ambulation were encouraged for all patients. Patients with clinically suspected deep vein thrombosis or a pulmonary venous thromboembolic event (VTE) were sent for further investigation. Patients were discharged when they were able to walk with walking aid more than 10 m, pain was controlled by oral analgesics and there was no continuous bleeding or oozing from the wound.

The primary outcomes were: the volume of postoperative blood loss in the drain, changes in hemoglobin concentrations, and the necessity for a blood transfusion. As for secondary outcomes, we measured knee diameter for swelling, local soft tissue complications, skin necrosis, and clinically confirmed VTEs, and pain, using a Visual Analogue Scales (VAS, 0 = no pain, 10 = the worst imaginable pain), knee flexion at discharge and the length of hospital stay.

Knee swelling was measured circumferentially at the thigh (taken at 10 cm above the upper pole of the patella) and the lower leg (taken at 10 cm below the lower pole of the patella), and calculated as the differences in the circumferences pre- and 48-h post-operation.

### Sample size

The sample size was calculated based on the measured postoperative blood loss. We assumed an alpha error of 0.05 and applied an allocation ratio of 1. A sample size of 30 participants, which allowed with a dropout rate of 10 % (3 participants), was calculated to provide a 80 % power in detecting a difference of 150 mL or reducing postoperative blood loss by 30 % in favour of the peri-articular TXA injection (from 10 patients of our earlier pilot study); we considered these parameters to be clinically relevant [24]. All statistical analyses were performed using SPSS software version 17.0 (SPSS Inc, Chicago, IL, USA). Differences in categorical variables were analyzed by chi squared or Fisher’s exact test, as necessary, and the unpaired ‘t’ test was used for normally distributed, continuous variables.

## Results

Of the 63 patients seen, two were diagnosed with inflammatory arthritis and one patient with post-traumatic arthritis and so were excluded from the study. A total 60 patients were recruited, underwent a unilateral TKA and completed the study. Half received either peri-articular (group 1) or IV (group 2) TXA. Their ages ranged from 50–80 years. There were no significant differences preoperative Body Mass Index (BMI), hemoglobin, and hematocrit level between the two groups (Table 1).

**Table 1:** Patient characteristics at baseline

	Peri-articular group	Intravenous group	*P* value*
	(*n* = 30)	(*n* = 30)	
Sex (male/female)	5/25	7/23	-
Age (y)	67.63 (+/− 7.96)	69.97 (+/− 7.55)	0.248
Weight (kg)	66.88 (+/− 11.29)	65.14 (+/− 9.32)	0.517
Height (cm)	155.73 (+/− 6.59)	156.77 (+/− 6.39)	0.539
BMI (kg/m^2^)	27.96 (+/− 4.99)	26.52 (+/− 3.71)	0.208
Hemoglobin (g/dL)	12.01 (+/− 1.27)	12.27 (+/− 1.30)	0.436
Hematocrit (%)	36.58 (+/− 3.63)	37.58 (+/− 3.49)	0.283

Values are number or mean (+/−Standard deviation = SD)

*P < 0.05 considered significant

Blood loss measured in the drain postoperatively was less in group 1 at both time points but the difference was not statistically significant (Table 2). The changes in hemoglobin and hematocrit were small and similar between both groups as was the number of blood transfusions.

**Table 2:** Blood loss and blood transfusions

Variable	Group 1: Peri-articular group (*n* = 30)	Group 2: Intravenous group (*n* = 30)	*P* value*
Blood in Hemovac drain(ml)			
24-h postoperative	300 (+/− 128)	334 (+/− 124)	0.279
48-h postoperative	145 (+/− 92)	186 (+/− 106)	0.094
Total	445 (+/− 158)	520 (+/− 175)	0.081
Hematocrit (%)			
Preoperative	36.4 (+/− 3.6)	37.6 (+/− 3.5)	0.226
24-h postoperative	29.7 (+/− 3.4)	30.4 (+/− 4.0)	0.550
48-h postoperative	31.0 (+/− 2.7)	31.8 (+/− 3.4)	0.352
Total Hct change (%) (pre op – 48 hr)	−5.4 (+/− 3.1)	−5.8 (+/− 4.4)	0.730
Hemoglobin (g/dL)			
preoperative	12.01 (+/− 1.27)	12.27 (+/− 1.30)	0.628
24-h postoperative	9.69 (+/− 1.19)	10.09 (+/− 1.50)	0.215
48-h postoperative	10.16 (+/− 0.98)	10.40 (+/− 1.28)	0.693
Total Hb change (g/dL) (pre op – 48 hr)	−1.85 (+/− 0.95)	−1.87 (+/− 1.37)	0.840
Total blood transfusion (PRC in unit)	12	8	-
Number of patients with blood transfusion	9	7	0.928

Values are number or mean (+/−Standard deviation = SD)

*P < 0.05 considered significant

The postoperative leg, thigh swelling also showed no significant differences (Table 3).

**Table 3:** Thigh and leg circumferences (centimeters)

	Peri-articular group	Intravenous group	*P* value*
	(*n* = 30)	(*n* = 30)	
Thigh (cm)			
Preoperative	44.25 (+/− 6.61)	43.63 (+/− 4.24)	0.627
Postoperative	46.30 (+/− 7.03)	45.10 (+/− 4.21)	0.398
Difference	2.05 (+/− 1.47)	1.47 (+/− 1.19)	0.093
Leg (cm)			
Preoperative	35.20 (+/− 3.88)	33.57 (+/− 2.97)	0.069
Postoperative	36.57 (+/− 3.96)	34.70 (+/− 3.37)	0.052
Difference	1.39 (+/− 1.27)	1.16 (+/− 1.08)	0.491

Values are number or mean (+/−Standard deviation = SD)

*P < 0.05 considered significant

VAS for pain at the 24 and 48 h assessments, postoperative knee flexion at discharge and hospital lengths of stay were similar (Table 4).

**Table 4:** Visual Analogue score (VAS) for pain, knee flexion at discharge, and length of hospital stay

	Peri-articular group	Intravenous group	*P* value*
	(*n* = 30)	(*n* = 30)	
VAS (0–10)			
24-h postoperative	3.8 (+/− 2.48)	3.70 (+/− 2.44)	0.889
48-h postoperative	3.56 (+/− 1.89)	3.43 (+/− 1.67)	0.724
Knee flexion at discharge	97° (+/− 10.87°)	90° (+/− 17.41°)	0.087
Hospital stay(Days)	5.7 (+/− 1.46)	5.3 (+/− 0.84)	0.276

Values are number or mean (+/−Standard deviation = SD)

*P < 0.05 considered significant

No patients had clinical evidence of tense hemarthroses, subcutaneous hematomas, peroneal nerve palsies, surgical wound infections, skin necrosis or symptomatic VTEs up to 14 days following the surgery.

## Discussion

TXA has been shown to be an effective antifibrinolytic agent for reducing blood loss following TKA [8, 10]. However, IV TXA is a systemic therapy and requires systemic distribution to exert its antibleeding effects. Peri-articular TXA injection could, theoretically, be more effective and cause less systemic toxicity like thrombosis and systemic hypersensitivity reactions [25]. Given the lack of data, a randomized trial was needed comparing peri-articular with the current standard treatment of IV TXA.

Our study has demonstrated that the 750 mg of peri-articular TXA injection group had less blood loss in the drain and lower decreases in hemoglobin concentration but a slight increase in the number of blood transfusion rates which would be from lower initial hemoglobin level in the peri-articular TXA injection group. Although none of these differences were statistically significant, the data hint that the antibleeding effects may have been roughly similar. This was a pilot study using a “best guess” dose. No dose ranging studies have been conducted to determine the optimal peri-articular dose and this is an area of future research.

There are the case reports of VTEs associated with IV TXA administration which is concern to the surgeon [16, 17]. Two recent meta-analyses of IV TXA in TKA was unable to conclude definitively on its safety because the studies were underpowered [26, 27]. Our study found no VTE in either group but our patient numbers were very small. The peri-articular TXA injection group did not show signs of drug toxicity as evidenced by the lack of local physical signs, like excess bleeding, swelling, and wound infections. This is a promising finding but more studies are needed, including dose ranging studies to ascertain if toxicity is dose related. Trying to detect if there might be differences in risk of VTEs between peri-articular and IV TXA would require very large sample sizes and is best left to pharmacovigilance if peri-articular TXA becomes an established practice. We acknowledge our study’s limitations. The sample size was small and so we were underpowered to detect uncommon events like VTEs. Moreover, we were guided by symptoms and signs of VTEs and did not actively seek asymptomatic VTEs. A lack of resources precluded the monitoring of serum TXA concentrations which could have suggested under or over exposure of IV TXA. We did not establish placebo group in the study, since the administration of IV TXA is a standard protocol for our patients and possible problem with ethical issues. The previous prospective randomized controlled trial study comparing IV TXA with placebo in the patients undergone TKA found significantly lower blood loss in IV TXA group [13].

## Conclusions

Our small pilot study has shown promising initial results regarding peri-articular TXA injection. This, in primary TKA, peri-articular TXA injection could be an alternative route of TXA administration. More research is needed to determine the optimal TXA dose and define better the risks and benefits.

## Abbrevations

BMI, body mass index; TKA, total knee arthroplasty; VAS, visual analogue scales for pain; VTE, venous thromboembolic event
